# Takotsubo Cardiomyopathy: A Consequence of Blunt Chest Trauma

**DOI:** 10.7759/cureus.78745

**Published:** 2025-02-08

**Authors:** Aditya Lal Vallath, Michael Galuska, Taylor Campbell

**Affiliations:** 1 Emergency Medicine, Duke Lifepoint Conemaugh Memorial Medical Center, Johnstown, USA

**Keywords:** blunt trauma chest, emergency medicine, pericarditis, pocus, takotsubo cardiomyopathy (ttc)

## Abstract

Takotsubo cardiomyopathy (TTC), also known as stress-induced cardiomyopathy or "broken heart syndrome," is a transient form of myocardial dysfunction often triggered by emotional or physical stress. While typically associated with emotional distress and possible physical stress, TTC has rarely been reported in the context of physical trauma, including blunt chest injuries. This case report describes a novel instance of TTC in an 84-year-old woman who developed the condition following a motor vehicle accident, resulting in blunt chest trauma. Coronary artery disease was ruled out through coronary angiography, which showed no significant coronary obstruction. The patient’s left ventricular ejection fraction at presentation was 25-30%, indicative of severe systolic dysfunction. The patient's clinical course, diagnostic findings, and management are discussed, contributing to the literature by expanding the understanding of TTC’s potential occurrence following blunt trauma. This case supports prior findings that emotional and physical stress can trigger TTC but challenges the assumption that TTC is predominantly linked to emotional triggers. It also introduces a novel aspect of TTC in the elderly, emphasizing the importance of considering this diagnosis in patients presenting with chest pain and cardiac dysfunction after physical trauma.

## Introduction

Takotsubo cardiomyopathy (TTC), also known as stress-induced cardiomyopathy or "broken heart syndrome," is a transient form of myocardial dysfunction often triggered by emotional or physical stress. Although TTC is more commonly linked to emotional stress or physical stress, it can occur, albeit rarely, after physical trauma, including blunt chest injuries. The proposed mechanisms in these cases involve a surge in catecholamines or autonomic dysregulation, both of which can precipitate transient myocardial dysfunction following significant physical stress [1]. This case report presents a particularly novel instance of TTC in an 84-year-old woman who developed the condition after a motor vehicle accident (MVA) resulting in blunt chest trauma. What makes this case unique is not only the patient's age but also the severity of systolic dysfunction, with an initial left ventricular ejection fraction (LVEF) of 25-30%, indicating severe impairment. Coronary artery disease was ruled out through coronary angiography, which showed no significant coronary obstruction. The patient's clinical course, diagnostic findings, and management are discussed, highlighting the importance of considering TTC in elderly patients presenting with chest pain and cardiac dysfunction following blunt trauma. This case contributes to the literature by illustrating how physical trauma, particularly in the elderly, can trigger TTC, challenging the assumption that the condition is predominantly linked to emotional stress.

## Case presentation

An 84-year-old woman with a past medical history of hyperlipidemia and hypothyroidism presented to the emergency department, after being involved in an MVA. She was the driver of a vehicle that collided with another car, resulting in airbag deployment and blunt chest trauma. Although initially evaluated by EMS at the scene, she declined transport to the hospital and went home. However, later in the day, she sought medical attention due to chest discomfort radiating to her back. She denied difficulty breathing, numbness, weakness, or any other neurological symptoms. On examination, the patient displayed bruising across her chest, but there were no signs of acute respiratory distress or other traumatic injuries beyond minor abrasions and a small hematoma on her left thumb. Her initial findings are noted in Table 1.

**Table 1:** Initial findings on presentation HENT: Head, eyes, nose, throat; MCP: metacarpophalangeal; JVD: jugular vein distention

Initial findings on presentation	
Physical Exam	
Vitals	Temp: 37.1°C (98.7°F), HR: 106, Resp: 20, BP: 151/89, SpO2: 99%
Constitutional	Well appearing, no apparent distress
Eyes	Pupils equal, round, reactive to light, no conjunctival pallor
HENT	Mucous membranes moist, oropharynx unremarkable
Cardiovascular	Heart regular, no murmurs, good peripheral pulses
Pulmonary/Chest	Lungs clear, no wheezing, rales, or rhonchi, no respiratory distress
Abdominal	Soft, nontender, nondistended, normal bowel sounds, no guarding or rigidity, no pulsatile mass
Musculoskeletal	Left first MCP joint discomfort (no deformity), 4-inch subcutaneous hematoma on chest, no crepitus or JVD
Neurologic	AAOx3, no focal deficits, mild memory issues related to bruises
Skin	Superficial bruises on shins, no deformity or joint effusion, normal sensation and strength
Labs	
CBC	
	WBC: 10.7 (3.10 - 8.50 10*3/uL)
	Hemoglobin: 14.5 (11.5 - 16.0 g/dL)
	Platelets: 241 (140 - 440 10*3/uL)
Basic Metabolic Panel	Sodium: 135 (136 - 145 mmol/L)
	CO2: 20 (22 - 29 mEq/L)
	Creatinine: 1.12 (0.60 - 1.10 mg/dL)
	eGFR: 49 (>60 mL/min)
	Glucose: 136 (83 - 110 mg/dL)
Troponin I	Troponin I: 3.43 (0.00 - 0.05 ng/mL)
Coagulation Studies	APTT: 23 (23 - 35 seconds)
	Protime: 9.9 (9.0 - 12.0 seconds)
	INR: 1.0 (normal)
Type & Screen	ABO/Rh: A positive
	Antibody Screen: Negative
Radiology	
CT Chest	No acute injury to thorax, clear lungs, no pneumothorax or pleural effusion, no fracture, coronary artery calcification
CT Abdomen/Pelvis	No visceral or bony injury, multiple colonic diverticula, small fat-containing umbilical hernia, normal organs
X-ray	No evidence of fractures or dislocations noted

**Figure 1:**
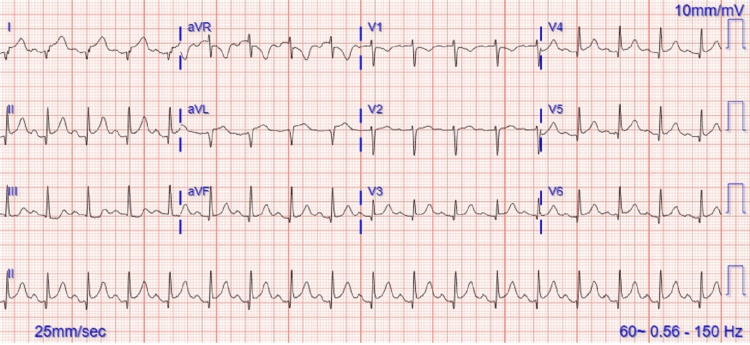
ECG on the initial presentation showing diffuse sinus tachycardia ST elevation

Her ECG (electrocardiogram) done at triage on initial presentation is noted in Figure 1. 

Upon arrival at the level 1 trauma center, the patient denied dizziness, dyspnea on exertion, shortness of breath, or loss of consciousness but complained of localized chest pain at the site of bruising on the anterior chest wall. On examination, she had tenderness in the left anterior chest wall and mild tenderness in the lower abdomen. No fractures or open wounds were observed. The patient's concerns were addressed, and analgesia was provided for comfort. An EFAST (extended focused assessment with sonography for trauma) scan was conducted which revealed mild pericardial fluid; however, no other signs of trauma such as free fluid in the abdomen or pneumothorax were noted. Chest and abdominal CT scans with contrast were performed, revealing no visceral or bony injuries, but mild atelectasis in the lungs and a small cyst in the liver were noted. X-rays of the limbs showed no fractures or dislocations. While a STEMI (ST elevated myocardial infarction) was initially suspected due to widespread ST elevation on the ECG, the cardiologist also considered blunt trauma-induced pericarditis given the non-reciprocal ST changes. However, given that a STEMI could not be definitively ruled out, heparin infusion therapy was initiated as a precautionary measure. The patient’s high-sensitivity troponin levels were elevated at 3.43ng/ml, and she was admitted to the ICU for close monitoring. An echo was scheduled for the following morning to further assess the patient's cardiac function. 

A summary of her clinical findings can be found in Table 2.

**Table 2:** Patient's physical, clinical, and radiological findings at presentation at our trauma center

Category	Details
Vital Signs	
Temperature	36.7°C (98.1°F)
Heart Rate	101 bpm
Respiratory Rate	18 bpm
Blood Pressure	118/77 mmHg
SpO2	97%
Weight	125 lbs (56.7 kg)
Pain Score	4 (Moderate pain)
Body System	Findings
General	Well-appearing, no distress
Cardiovascular	Regular heart rhythm, no murmurs, good peripheral pulses
Pulmonary/Chest	Clear lungs bilaterally, bruising on left upper chest, tenderness on left anterior chest wall, no rib deformity or open wounds
Abdominal	Longitudinal bruising (seatbelt sign), no peritonitis, no hepatosplenomegaly
Musculoskeletal	No deformities, no pedal edema
Neurologic	Alert, oriented, no focal deficits
Skin	Warm, well-perfused, no acute rashes
Psychiatric	Normal affect
Laboratory Results	
Troponin I	11,415 ng/L (high sensitivity)
Amylase	123 U/L
CBC	WBC: 10.51 x 10^3 (3.10 - 8.50 x 10^3/uL)
	Hemoglobin: 13.0 (11.5 - 16.0 g/dL)
	Platelets: 206 (140 - 440 x 10^3/uL)
Basic Metabolic Panel	Sodium: 135 (136 - 145 mmol/L)
	CO2: 22 (23 - 31 mEq/L),
	eGFR: 50 (>60 mL/min)
	Glucose: 131 mg/dL (Fasting 83 - 110 mg/dL)
	Potassium: 4.0 (3.5 - 5.1 mmol/L)
	Chloride: 104 (98 - 107 mmol/L)
	Creatinine: 1.10 (0.60 - 1.10 mg/dL)
	Magnesium: 2.2 (1.6 - 2.6 mg/dL)
	Phosphorus: 3.7 (2.3 - 4.7 mg/dL)
	Lactate: 1.4 (0.5 - 2.0 mmol/L)
Coagulation Studies	aPTT: 23 s (23 - 35 Seconds)
	Protime: 10.9 (9.0 - 12.0 seconds)
	INR: 1.0 (0.8 - 1.1)
E-FAST Scan	
Thorax	Good lung sliding in all quadrants, no evidence of pneumothorax
Heart	Small pericardial effusion, no evidence of right ventricular collapse
Hepatorenal Pouch	No evidence of free fluid noted
Splenorenal Pouch	No evidence of free fluid noted
Pelvis	No evidence of free fluid around the bladder
Radiological Findings	
CT Abdomen & Pelvis	No visceral or bony injury, small umbilical hernia, multilevel lumbar disc protrusions
CT Chest	Clear lungs, no pneumothorax or pleural effusion, no aortic injury, coronary artery calcification
X-rays	No fractures or dislocations in tibia, fibula, ankle, wrist, or knees; osteoarthritis noted in the left wrist

The patient was assessed by the surgical trauma team who after careful assessment found no acute traumatic injuries and recommended admitting the patient in the ICU for medical management. In the ICU, the patient was managed with close monitoring, heparin infusion, and measures to trend troponin levels. There were no acute concerns regarding respiratory, gastrointestinal, or renal systems. The plan included NPO status, monitoring vital signs, and adjusting glucose levels as needed. 

On day 2, the patient was treated symptomatically for mild pain; however, there was no acute deterioration. Her heparin infusion was continued as protocol. Her initial troponin was elevated at 11,000, trending down to 9,755, with CK and myoglobin also elevated. Her lab values in the first 48 hours are reported in Table 3. 

**Table 3:** Lab values in the first 48 hours

Lab Component	Units	Day 1	Day 2	Range
WBC	10³/µL	7.74	N/a	4.50 - 11.00 10*3/uL
Hemoglobin	g/dL	13.0	N/a	11.5 - 16.0 g/dL
Hematocrit	%	39	N/a	37 - 47 %
Platelets	10³/µL	222	N/a	140 - 440 10*3/uL
Sodium	mmol/L	137	N/a	136 - 145 mmol/L
Potassium	mmol/L	4.1	N/a	3.5 - 5.1 mmol/L
Chloride	mmol/L	104	N/a	98 - 107 mmol/L
CO2	mEq/L	23	N/a	22 - 29 mEq/L
BUN	mg/dL	18	N/a	9 - 21 mg/dL
Creatinine	mg/dL	1.00	N/a	0.60 - 1.10 mg/dL
Glucose (Fasting)	mg/dL	121	N/a	70 - 105 mg/dL
Calcium	mg/dL	9.10	N/a	8.50 - 10.30 mg/dL
Troponin I HS	ng/L	9,755	10,716	0 - 34 ng/L
CK Total	U/L	N/a	542	30 - 190 U/L
Troponin I	ng/mL	N/a	3.43	0.00 - 0.05 ng/mL

The decision was taken to undergo transthoracic echocardiography by the cardiologist in order to assess for the possible presence of cardiac dysfunction in this patient. Echocardiography revealed a left ventricle with normal size but mild thickening of the heart muscle (concentric hypertrophy). Severe systolic dysfunction was observed with a significantly reduced ejection fraction (25-30%). Diastolic filling was not assessed, and left atrial pressure remained within normal limits. Several segments of the left ventricle showed reduced or no movement (akinesis) as well as the presence of apical ballooning was noted on the echo. The right ventricle exhibited normal size and function. Both atria were of normal size. The inferior vena cava was dilated and demonstrated poor collapse, suggesting elevated right atrial pressure. Mild regurgitation was noted on the mitral, tricuspid, and aortic valves, with no significant narrowing (stenosis). The pulmonic valve appeared normal, but pulmonary artery pressure was moderately elevated at 46 mmHg, indicative of moderate pulmonary hypertension. A small pericardial effusion was present without evidence of cardiac tamponade.

An echo video noted in Video 1 represents the parasternal long-axis view. Note the apical dilation in the left ventricle. 

**Video 1:** The parasternal long-axis view with the apical dilation in the left ventricle

Based on the findings of the echocardiogram, a decision was made to catheterize the patient to rule out ischemic etiology. Informed consent was obtained from the patient after a thorough discussion of the procedure. Following sterile preparation and local anesthesia, a diagnostic coronary angiogram was performed via right radial artery access. Left and right coronary arteries were visualized and found to be normal with the exception of a non-occlusive myocardial bridge in the mid-left anterior descending artery (LAD). Left ventriculography revealed severe systolic dysfunction with an ejection fraction of approximately 25%. Regional wall motion abnormalities consistent with apical ballooning (Takotsubo cardiomyopathy) were observed. Left ventricular end-diastolic pressure was normal at 9 mmHg. Hemodynamic assessment across the aortic valve revealed no significant pressure gradient. Coronary artery catheterization is noted in Video 2.

**Video 2:** Coronary catheterization

In conclusion, the patient had non-obstructive coronary artery disease and TTC. The patient was managed conservatively and was started on metoprolol 12.5 mg daily. On day 3, she was found to be comfortable. She was discharged with a LifeVest® wearable cardioverter defibrillator (WCD) and advised to continue her previous medications as well as metoprolol 12.5 mg daily. The WCD and metoprolol were prescribed to her as a precautionary measure against cardiac arrhythmias. She was also advised to follow up with her cardiologist and primary care provider for close monitoring. Her final diagnosis on discharge was TTC and pericardial effusion secondary to blunt chest injury from an MVA. 

## Discussion

TTC, also known as stress-induced cardiomyopathy or "broken heart syndrome," is a transient cardiac condition typically triggered by severe emotional or physical stress. It presents with symptoms similar to acute myocardial infarction, including chest pain and ST-segment elevation on ECG, but without the presence of obstructive coronary artery disease [1]. The diagnosis of TTC often relies on the modified Mayo Clinic criteria, which include the absence of significant coronary artery disease on angiography, transient abnormalities in left ventricular wall motion with mid-segment hypokinesis or akinesis and potential apical involvement, electrocardiographic evidence of ST-segment elevation and/or T-wave inversion, modest elevations in cardiac troponin levels, and the exclusion of other conditions such as myocarditis and pheochromocytoma [2]. The pathophysiology is thought to involve a sudden surge of catecholamines, leading to myocardial stunning, particularly affecting the left ventricle. This results in characteristic findings on imaging, such as a severely reduced ejection fraction with regional wall motion abnormalities, most commonly in the apical and mid-ventricular regions, while sparing the basal segments. This was first described by Sato et al. in 1990 after which the term was coined as Takatsubo cardiomyopathy after the classic octopus traps [3].

In this case, the patient's left ventriculogram revealed a 25% ejection fraction with anterolateral, apical, and diaphragmatic akinesis, supporting the TTC diagnosis. While a mid-LAD myocardial bridge could theoretically exacerbate stress-induced myocardial dysfunction in TTC by contributing to intermittent ischemia, in this case, it was non-occlusive. TTC, triggered by intense emotional or physical stress, causes temporary heart muscle weakening, particularly in the left ventricle. Although often self-limiting and requiring supportive care, recognizing TTC is crucial for appropriate management and avoiding unnecessary interventions. The etiology of TTC remains incompletely understood but is believed to involve catecholamine surges triggered by acute stress, leading to transient left ventricular dysfunction, particularly in the apical and mid-ventricular regions [4]. Additional contributing factors may include microvascular spasm, endothelial dysfunction, and inflammation. Risk factors include emotional stress, physical trauma, neurological events, or severe illness, with postmenopausal women being more commonly affected [4].

The exact cause of the contractile pattern in TTC is unclear, with several theories proposed. One suggests multivessel coronary artery spasm may lead to regional myocardial stunning, but this does not explain the severe apical dysfunction or mild cardiac enzyme elevation [3,5-7]. While coronary microvascular impairment has been observed, its role remains uncertain. Abnormal left ventricular wall motion, particularly affecting the apical myocardium, suggests potential disturbances in the coronary microcirculation. Studies, including those by Kume et al., Yoshida et al., and Khalid et al., have shown impaired coronary perfusion and metabolic abnormalities [8-10], with Elesber et al. noting a correlation between microvascular dysfunction and the severity of myonecrosis and ECG changes [11]. These findings indicate microvascular abnormalities may be involved, but whether they are a cause or result of apical ballooning remains unclear [12].

Elevated catecholamine levels, particularly epinephrine, appear to trigger myocardial dysfunction in TTC [13-15]. These high levels can lead to intracellular calcium overload, contraction band necrosis, and myocardial stunning. Elevated catecholamines (norepinephrine, epinephrine, and dopamine) are consistently observed in TTC patients, often two to three times normal [13-15]. This catecholamine surge induces changes in myocardial contractility via beta-2-adrenoceptor stimulation, leading to negative inotropic effects and left ventricular dysfunction. This "stimulus trafficking" is particularly relevant in the apical form of TTC, where beta-adrenergic receptors are most concentrated. Intravenous catecholamine or beta-adrenergic agonist administration can reproduce TTC's clinical features, further supporting the role of excessive catecholamine release [13-15]. Beta-blockers, such as metoprolol, reduce myocardial oxygen demand and myocardial stunning by blocking β1 and β2 receptors. Thus, the rationale behind using beta-blockers in TTC is to mitigate the acute effects of catecholamine excess on the heart, stabilizing the patient’s condition and helping prevent further myocardial damage or arrhythmias. While there is no definitive consensus on the optimal timing and duration for beta-blocker therapy in TTC, it is postulated that their use during the acute phase provides symptomatic relief and supports recovery by reducing stress on the heart muscle and minimizing the risk of further complications. As such, beta-blockers have become a key component in the management of TTC, with the catecholamine hypothesis being widely accepted as the primary pathophysiologic mechanism [13-15].

TTC's pathophysiology remains complex. Histologically, it is characterized by interstitial infiltrates (primarily mononuclear lymphocytes, leukocytes, and macrophages), myocardial fibrosis, and contraction bands, distinguishing it from ischemic myocardial infarction [16]. Inflammation is believed to play a significant role. Cardiac MRI reveals myocardial edema, necrosis, and fibrosis [16]. Late gadolinium enhancement (LGE), while once thought absent, is present in up to 10% of cases, typically in a focal or patchy pattern [17,18], distinct from myocarditis or ischemia. This LGE usually resolves with follow-up. Studies have shown macrophage recruitment, monocyte subtype changes, and increased pro-inflammatory cytokines [19]. Coexisting inflammatory or autoimmune conditions have been reported [20], though diagnostic guidelines (Mayo Clinic and the European Society of Cardiology) exclude myocarditis as a TTC criterion. Histological analysis has shown contraction band necrosis, inflammatory cell infiltration, and focal fibrosis, possibly from catecholamine cardiotoxicity [21].

Estrogen has cardioprotective effects, including vasodilation and prevention of atherosclerosis and endothelial dysfunction [22]. The predominance of postmenopausal women among TTC patients suggests estrogen deficiency may increase the risk. Studies show that the absence of estrogen replacement therapy may predispose women to the condition, and murine models indicate that ovariectomy removes cardiac protection, which is restored with estradiol [23]. Estrogen may also down-regulate beta-adrenergic receptors, linking its deficiency to Takotsubo risk. While rarer in men, TTC tends to have a worse prognosis in males, potentially due to the lack of estrogen’s protective effects [24].

The "aborted myocardial infarction" hypothesis suggests Takotsubo syndrome results from transient coronary occlusion due to acute thrombus formation followed by rapid lysis. Though angiography is often normal, intravascular imaging (IVUS, OCT) has revealed atherosclerotic vulnerabilities, such as eccentric plaques and thin-cap fibroatheromas in the mid-LAD, which may lead to transient occlusion [25,26]. Coronary artery vasospasm has also been implicated, with studies showing evidence of spasm and vasoconstriction, supported by acetylcholine provocation testing [3,27].

Neurogenic mechanisms, such as central nervous system activation during emotional distress, may also play a role in the myocardial changes seen in TTC. Overall, the condition is likely due to a combination of high local catecholamine concentrations, myocardial necrosis, and impaired adrenergic responsiveness, primarily induced by severe emotional stress. A combination of factors as discussed above may be responsible for the development of TTC in this patient. Trauma as a cause has been rarely implicated in the development of TTC. Ahmed et al. reported a case with reverse TTC and commotio cordis secondary to trauma from an MVA. The case was reported to be a variant of TTC which showed basal hypokinesis/akinesis with apical sparing or hyperkinesis [28]. Another case report by Ritchie et al. reported a 59-year-old woman who developed TTC and pulmonary edema following a motor vehicle collision, presenting with respiratory distress and ST elevation on ECG, but without coronary artery occlusion. Coronary angiography confirmed apical ballooning, and the patient improved rapidly. TTC poses a diagnostic challenge, especially in trauma patients where other serious conditions, such as acute coronary syndrome (ACS) or aortic injury, must be ruled out. A systematic review by Gruhl et al. found that patients with traumatic brain injury may develop TTC due to catecholamine excess. It was reported that these patients underwent quick resolution of cardiac pathology as long as the TBI was managed appropriately [29]. Similarly, Cimaroli et al. demonstrated a case of TTC similar to this case in a patient following blunt chest trauma [30] which highlights the need for further evaluation to assess trauma as a potential cause for it.

The differential diagnosis for TTC is broad, encompassing several conditions that can mimic ACS. A key challenge is differentiating TTC from myocardial infarction (MI), as both can present with chest pain, ECG changes, and elevated cardiac enzymes. However, TTC is typically characterized by the absence of obstructive coronary artery disease on angiography and the presence of left ventricular apical ballooning. Myocardial contusion, particularly in cases of trauma, is another important consideration. It can also cause chest pain and ECG abnormalities. However, unlike TTC, myocardial contusion often shows evidence of direct myocardial injury, such as wall motion abnormalities that extend beyond the apex and may be associated with other traumatic injuries. Other conditions that should be considered include myocarditis, pericarditis, and pulmonary embolism, each with distinct clinical and diagnostic features. To differentiate TTC from these other conditions, a comprehensive evaluation involving cardiac imaging and biomarker testing is essential. TTC typically has an excellent prognosis with nearly full recovery within 6-8 weeks. Recurrence rates are low, estimated at around 1-2%. Mortality rates are generally low, reported to be between 3-4%. However, complications can occur in a significant proportion of patients (at least 25%). These complications may include mitral regurgitation (mild to moderate), left heart failure, cardiogenic shock, dynamic left ventricular outflow tract obstruction, the development of left ventricular mural thrombi, ventricular arrhythmias, ventricular wall rupture, and, in rare cases, death [4]. 

## Conclusions

In conclusion, this case underscores the importance of considering TTC in patients presenting with acute coronary syndrome-like symptoms following significant trauma, such as an MVA. "Apparent myocardial injury" in this context refers to the absence of both coronary artery occlusion (as would be seen in a myocardial infarction) and evidence of myocardial necrosis on initial imaging. The stress of the traumatic event can precipitate catecholamine-induced myocardial dysfunction, leading to the characteristic apical ballooning of TTC, even without direct cardiac injury. Differentiating TTC from other life-threatening conditions, particularly myocardial infarction, is crucial to avoid unnecessary interventions such as thrombolysis or coronary angioplasty. Misdiagnosis as ACS could also lead to inappropriate administration of antithrombotic therapy, with its associated risks. This case demonstrates the generally favorable prognosis of TTC with appropriate recognition and supportive care. However, while most patients experience rapid recovery, it is important to acknowledge the potential for complications such as heart failure, arrhythmias, or cardiogenic shock. A comprehensive diagnostic approach, integrating advanced imaging like cardiac MRI and biomarker assessment (including troponins and BNP), is essential. Finally, appropriate follow-up care is necessary to monitor for recurrence or the development of long-term myocardial dysfunction such as heart failure or cardiomyopathy.

## References

[1] Ahmad SA, Brito D, Khalid N, Ibrahim MA (2025). Takotsubo cardiomyopathy. StatPearls [Internet].

[2] Prasad A, Lerman A, Rihal CS (2008). Apical ballooning syndrome (Tako-Tsubo or stress cardiomyopathy): a mimic of acute myocardial infarction. Am Heart J.

[3] Sato H, Tateishi H, Uchida T (1990). Takotsubo-type cardiomyopathy due to multivessel spasm. Clinical Aspect of Myocardial Injury: From Ischemia to Heart Failure.

[4] Khalid N, Ahmad SA, Shlofmitz E, Chhabra L (2024). Pathophysiology of Takotsubo syndrome. StatPearls [Internet].

[5] Inoue M, Shimizu M, Ino H (2005). Differentiation between patients with takotsubo cardiomyopathy and those with anterior acute myocardial infarction. Circ J.

[6] Bybee KA, Motiei A, Syed IS (2007). Electrocardiography cannot reliably differentiate transient left ventricular apical ballooning syndrome from anterior ST-segment elevation myocardial infarction. J Electrocardiol.

[7] Nef HM, Mollmann H, Weber M, Deetjen A, Brandt R, Hamm CW, Elsasser A (2007). Release pattern of cardiac biomarkers in left ventricular apical ballooning. Int J Cardiol.

[8] Kume T, Akasaka T, Kawamoto T (2005). Assessment of coronary microcirculation in patients with takotsubo-like left ventricular dysfunction. Circ J.

[9] Yoshida T, Hibino T, Kako N (2007). A pathophysiologic study of tako-tsubo cardiomyopathy with F-18 fluorodeoxyglucose positron emission tomography. Eur Heart J.

[10] Khalid N, Ahmad SA, Umer A (2016). Coronary flow reserve assessment via invasive and noninvasive means in Takotsubo cardiomyopathy. Int J Cardiol.

[11] Elesber A, Lerman A, Bybee KA (2006). Myocardial perfusion in apical ballooning syndrome correlate of myocardial injury. Am Heart J.

[12] Khalid N, Ikram S (2015). Microvascular dysfunction in Takotsubo cardiomyopathy: prognostic implications. Int J Cardiol.

[13] Wittstein IS, Thiemann DR, Lima JA (2005). Neurohumoral features of myocardial stunning due to sudden emotional stress. N Engl J Med.

[14] Lyon AR, Rees PS, Prasad S, Poole-Wilson PA, Harding SE (2008). Stress (Takotsubo) cardiomyopathy--a novel pathophysiological hypothesis to explain catecholamine-induced acute myocardial stunning. Nat Clin Pract Cardiovasc Med.

[15] Paur H, Wright PT, Sikkel MB (2012). High levels of circulating epinephrine trigger apical cardiodepression in a β2-adrenergic receptor/Gi-dependent manner: a new model of Takotsubo cardiomyopathy. Circulation.

[16] Eitel I, von Knobelsdorff-Brenkenhoff F, Bernhardt P (2011). Clinical characteristics and cardiovascular magnetic resonance findings in stress (takotsubo) cardiomyopathy. JAMA.

[17] Eitel I, Lücke C, Grothoff M, Sareban M, Schuler G, Thiele H, Gutberlet M (2010). Inflammation in takotsubo cardiomyopathy: insights from cardiovascular magnetic resonance imaging. Eur Radiol.

[18] Fazzini L, Casula M, Cau R (2024). The detection rate of late gadolinium enhancement in Takotsubo syndrome: a systematic review and meta-analysis. Am J Cardiol.

[19] Scally C, Abbas H, Ahearn T (2019). Myocardial and systemic inflammation in acute stress-induced (Takotsubo) cardiomyopathy. Circulation.

[20] Chhabra L, Khalid N, Kluger J, Spodick DH (2014). Lupus myopericarditis as a preceding stressor for takotsubo cardiomyopathy. Proc (Bayl Univ Med Cent).

[21] Lyon AR, Bossone E, Schneider B (2016). Current state of knowledge on Takotsubo syndrome: a position statement from the Taskforce on Takotsubo Syndrome of the Heart Failure Association of the European Society of Cardiology. Eur J Heart Fail.

[22] Moolman JA (2006). Unravelling the cardioprotective mechanism of action of estrogens. Cardiovasc Res.

[23] Kuo BT, Choubey R, Novaro GM (2010). Reduced estrogen in menopause may predispose women to takotsubo cardiomyopathy. Gend Med.

[24] Khalid N, Ahmad SA, Umer A, Chhabra L (2019). Factors impacting prognosis among patients with Tako-tsubo syndrome. Rev Esp Cardiol (Engl Ed).

[25] Ibanez B, Navarro F, Cordoba M, M-Alberca P, Farre J (2005). Tako-tsubo transient left ventricular apical ballooning: is intravascular ultrasound the key to resolve the enigma?. Heart.

[26] Shlofmitz E, Kerndt CC, Parekh A, Khalid N (2024). Intravascular ultrasound. StatPearls [Internet].

[27] Angelini P (2008). Transient left ventricular apical ballooning: a unifying pathophysiologic theory at the edge of Prinzmetal angina. Catheter Cardiovasc Interv.

[28] Ahmed Y, Rafique M, Ahmad S, Omar B, Malozzi C (2022). Reverse Takotsubo cardiomyopathy in a patient with commotio cordis. J Med Cases.

[29] Gruhl SL, Su J, Chua WC, Tay KV (2022). Takotsubo cardiomyopathy in post-traumatic brain injury: a systematic review of diagnosis and management. Clin Neurol Neurosurg.

[30] Cimaroli S, Maniar Y, Ciancarelli J, Stright A, Joseph D (2023). Takotsubo cardiomyopathy following blunt trauma: early recognition and diagnosis. Trauma Case Rep.

